# Sequencing and analysis of gerbera daisy leaf transcriptomes reveal disease resistance and susceptibility genes differentially expressed and associated with powdery mildew resistance

**DOI:** 10.1186/s12870-020-02742-4

**Published:** 2020-11-30

**Authors:** Krishna Bhattarai, Ana Conesa, Shunyuan Xiao, Natalia A. Peres, David G. Clark, Saroj Parajuli, Zhanao Deng

**Affiliations:** 1 Department of Environmental Horticulture, Gulf Coast Research and Education Center, University of Florida, IFAS, 14625 County Road 672, Wimauma, FL 33598 USA; 2 Department of Microbiology and Cell Science, University of Florida, IFAS, Gainesville, FL 32611 USA; 3 University of Florida, Genetics Institute, Gainesville, FL 32611 USA; 4 University of Maryland, College of Agriculture and Natural Resources, 4291 Fieldhouse Drive, Rockville, MD 20850 USA; 5 Department of Plant Pathology, Gulf Coast Research and Education Center, University of Florida, IFAS, 14625 County Road 672, Wimauma, FL 33598 USA; 6 Department of Environmental Horticulture, University of Florida, IFAS, Gainesville, FL 32611 USA

**Keywords:** Differentially expressed genes, Disease resistance, Gerbera, Powdery mildew resistance, *R*-gene, Single nucleotide polymorphisms, Simple sequence repeats, Susceptibility gene

## Abstract

**Background:**

RNA sequencing has been widely used to profile genome-wide gene expression and identify candidate genes controlling disease resistance and other important traits in plants. Gerbera daisy is one of the most important flowers in the global floricultural trade, and powdery mildew (PM) is the most important disease of gerbera. Genetic improvement of gerbera PM resistance has become a crucial goal in gerbera breeding. A better understanding of the genetic control of gerbera resistance to PM can expedite the development of PM-resistant cultivars.

**Results:**

The objectives of this study were to identify gerbera genotypes with contrasting phenotypes in PM resistance and sequence and analyze their leaf transcriptomes to identify disease resistance and susceptibility genes differentially expressed and associated with PM resistance. An additional objective was to identify SNPs and SSRs for use in future genetic studies. We identified two gerbera genotypes, UFGE 4033 and 06–245-03, that were resistant and susceptible to PM, respectively. De novo assembly of their leaf transcriptomes using four complementary pipelines resulted in 145,348 transcripts with a N50 of 1124 bp, of which 67,312 transcripts contained open reading frames and 48,268 were expressed in both genotypes. A total of 494 transcripts were likely involved in disease resistance, and 17 and 24 transcripts were up- and down-regulated, respectively, in UFGE 4033 compared to 06–245-03. These gerbera disease resistance transcripts were most similar to the NBS-LRR class of plant resistance genes conferring resistance to various pathogens in plants. Four disease susceptibility transcripts (*MLO*-like) were expressed only or highly expressed in 06–245-03, offering excellent candidate targets for gene editing for PM resistance in gerbera. A total of 449,897 SNPs and 19,393 SSRs were revealed in the gerbera transcriptomes, which can be a valuable resource for developing new molecular markers.

**Conclusion:**

This study represents the first transcriptomic analysis of gerbera PM resistance, a highly important yet complex trait in a globally important floral crop. The differentially expressed disease resistance and susceptibility transcripts identified provide excellent targets for development of molecular markers and genetic maps, cloning of disease resistance genes, or targeted mutagenesis of disease susceptibility genes for PM resistance in gerbera.

**Supplementary Information:**

The online version contains supplementary material available at 10.1186/s12870-020-02742-4.

## Background

Gerbera daisy (*Gerbera hybrida*) is popular in the global floricultural trade for its wide array of bright colored flowers. It is predominantly grown as cut flower and increasingly as garden, bedding, patio and indoor plants. Commercial gerbera daisy originated from an artificial interspecific cross between two African species *G. viridifolia* and *G. jamesonii* giving rise to new color combinations, floral arrangements and ease to grow compared to the parental species [1]*.* Cut flower gerbera alone accounted for around €140 million at the Dutch auction in 2014 [2]; cut flower gerberas in the U.S. generated a wholesale value of $32 million in 2015 [3]. Thousands of gerbera cultivars have been released in the world, and significant genetic improvement has been achieved in certain traits such as flower number and vase life [4]. With an attractive and complex flower structure, gerbera has also been used as a model plant to study flower development in the Asteraceae family. It has also been used extensively in studies of plant secondary plant metabolism [5].

Powdery mildew (PM) (*Podosphaera xanthii* syn. *Sphaerotheca fusca*) is the most common and devastating disease in gerbera. The pathogen infects flowers, leaves and other plant parts, rapidly developing unsightly white powdery matt on the plant surfaces. This matt is comprised of mycelia and conidial structures and can severely limit plant growth, distort flowers, and cause plant death. The impact of PM is highly destructive in controlled environments and production structures like greenhouses and plastic tunnels, where the majority of gerbera production takes place. Control of PM in commercial production has heavily relied upon frequent use of fungicides. However, complete control with fungicides is difficult to achieve when environmental conditions are conducive to PM [6]. Development and use of host plant resistance (HPR) is considered to be a cost-effective, environmentally friendly strategy to control PM. HPR has been widely used in numerous other crops including wheat, barley, tomato, grapevine, cucumber, sunflower, and rose [7–14].

In gerbera, HPR for PM resistance conferred by *Pmr1* locus has been reported [15]. A different source but strong HPR to PM was identified in gerbera breeding lines UFGE 31–19 and UFGE 5–23, which have been used to develop new cultivars and genotypes, including UFGE 4033 [16, 17]). PM resistance (PMR) in UFGE 4033 has been confirmed in multi-year and multi-location trials. A previous study indicated that two linked loci, *Rpx1* and *Rpx2,* were involved in controlling the PMR in UFGE 4033 [16, 18]*.* Substantial advances have been made in *Arabidopsis* and several other plants toward understanding plant resistance to PM and cloning and characterization of PM resistance genes [11, 19–22]. However, genes or candidate genes underlying the PMR in gerbera are yet to be identified.

In recent years, next generation sequencing (NGS) technologies have greatly facilitated the development of genomic and transcriptomic resources in non-model species. Transcriptome sequencing or RNA sequencing (RNA-seq) based on NGS has become one of the most commonly used methods to understand genetic variation among genotypes with contrasting phenotypes. Previously, RNA-seq study for *Botrytis cinerea* resistance in gerbera identified 25 homologs involved in phenylpropanoid, flavonoid, ethylene and jasmonic acid pathways [23]. Using RNA-seq analysis and annotation, two NBS-LRR transcripts were identified at the *Pm21* locus in *Haynaldia villosa* [24]. In rose, RNA-seq in a PM (*Podosphaera pannosa*)-resistant species *Rosa longicuspis*, and a PM-susceptible species *R. gigantea* identified two candidate genes *RgMLO6* and *RgMLO7* involved in host-pathogen interaction [25].

Gerbera is highly heterozygous and has a relatively large genome (around 5.0 Gb) that is rich with repetitive elements. A gerbera reference genome has not been reported. For a long time, genomic resources in gerbera were limited to some expressed sequence tags (ESTs) [26]. Recently, RNA-seq studies have been conducted to understand gene expression associated with gerbera flower traits, stem bending and *Botrytis* resistance [23, 27]. So far, there has not been any study in gerbera to understand gene expression in response to PM. In this study, we screened seven gerbera breeding lines to select genotypes with contrasting resistance genotypes to PM and performed RNA-seq analysis of the selected genotypes during PM infection. The objectives of this study were 1) to characterize gerbera leaf transcriptome and understand its global gene expression profile, 2) to conduct differential expression analysis and identify transcripts involved in PM resistance, 3) to identify *Mildew Locus O*-like transcripts in gerbera, and 4) to discover single nucleotide polymorphisms (SNPs) and SSR markers present in the gerbera transcriptome.

## Results

### Powdery mildew susceptibility of gerbera breeding lines

**Fig. 1:**
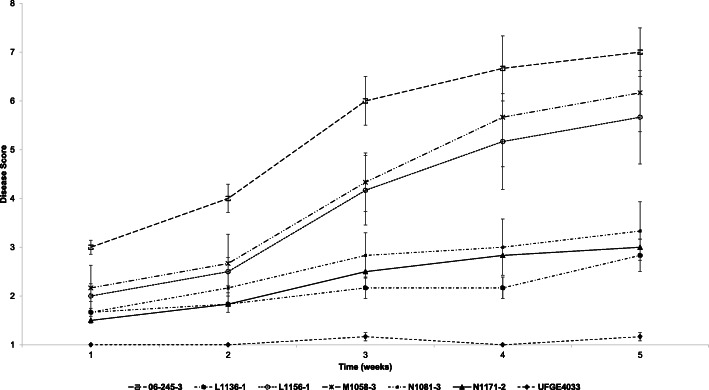
Screening of gerbera breeding lines for powdery mildew (PM) susceptibility. PM-resistant line UFGE 4033 was used as a control and a phenotypic rating scale of 1–10 where 1 indicates no or little visual presence of PM symptoms and 10 indicates the plant canopy completely covered with the disease. Data presented are means of three replicates. Error bars represent the standard error

Six PM-susceptible gerbera breeding lines, 06–245-03, M1058–3, L1156–1, N1081, N1171–2, L1136–1, and one PM-resistant breeding line, UFGE 4033, were evaluated for PM resistance in 2015–2016. Line 06–245-03 showed the highest level of PM susceptibility and was rated as the most susceptible among the selected lines with an AUDPC (Area Under Disease Progress Curve) score of 21.67 per week, whereas UFGE 4033 consistently showed few or no PM symptoms with an AUDPC score of 4.25 per week during the study (Fig. 1). Lines M1058–3, L1156–1, N1081, N1171–2 and L1136–1 were less susceptible, with AUDPC scores of 16.83, 15.67, 10.50, 9.42, and 8.42, respectively. Based on these data, breeding lines 06–245-03 and UFGE 4033 were selected as the most susceptible and the most resistant genotypes for RNA sequencing (Fig. S1). Typical PM symptoms observed in the gerbera lines are shown in Fig. S2.

### Transcriptome assembly

A total of 54.17 Gb of sequence data consisting of 180,559,609 paired-end (PE) reads were generated from sequencing three biological replicates of UFGE 4033 (93,675,701 PE reads) and 06–245-03 (86,883,908 PE reads) each (Table S1). PE reads resulted from sequencing the cDNA fragments from the both ends; this type of reads facilitates aligning sequence reads, construction of more accurate assemblies, and detection of novel transcripts. After removing the sequencing primers and barcodes from the raw reads and quality control, 51.80 Gb of cleaned sequencing data containing 172,658,001 (95.62%) cleaned reads were retained for further analysis. The average GC content of the cleaned reads was 46.84%. All cleaned reads, including 90,476,793 from UFGE 4033 and 82,181,208 from 06-245-03, were used for *de novo* assembly.

The final assembly constructed from the cleaned reads consisted of 145,348 transcripts (111,409,237 bp) with N50 of 1124 bp (Table 1). The smallest transcript was set at 125 bp, and there were 4903 transcripts that were smaller than 200 bp (Table 1). The mean length of transcripts was 761 bp, and the longest transcript contained 32,039 bp. Ten transcripts were longer than 10,000 bp, and 31,896 transcripts had lengths greater than 1000 bp. There were 67,312 transcripts with open reading fragments (ORFs), and the GC content of the assembly was 48.11% (Table 1).

**Table 1:** Major features of the *de novo* assembly of gerbera leaf transcriptome. Gerbera transcriptome was created from short read cDNA sequences of a powdery mildew-resistant (UFGE 4033) line and a susceptible (06–245-03) breeding line using four assemblers (Trinity, TransAbyss, SoapDenovo and Velvet pipelines)

Features	Values
Transcript distribution	
number of transcripts (no.)	145,348
smallest transcript (bases)	125
largest transcript (bases)	32,039
Total number of nucleotides	111,409,237
mean transcript length (bases)	761
transcripts under 200 bases	4903
transcripts over 1000 bases	31,896
transcripts over 10,000 bases	10
Transcripts with open reading frames (ORF)	67,312
mean ORF percent	81.21
Assembly length	
n90	358
n70	617
n50	1124
n30	1837

### Functional annotation

Among 145,348 transcripts submitted to Blast2GO, 75,390 (51.87%) transcripts had at least one BLASTX hit, 66,863 transcripts were mapped, and 46,258 transcripts were successfully annotated to other plant species (Fig. S3). Transcript functional annotation data are provided in Table S2. BLAST top-hits distribution indicated that the gerbera transcripts were most similar to *Helianthus annuus* and *Cynara cardunculus* var*. scolymus* (Fig. S4). Both *H. annuus* and *C. cardunculus* var. *scolymus* belong to the Asteraceae. The mapping procedure successfully mapped 66,863 (46.00%) transcripts. Gene ontology annotation of the mapped transcripts resulted in 46,258 (31.83%) transcripts to being successfully annotated (Fig. S5). Further, 74,195 GO-terms were categorized in WEGO2.0 [28] of which 54,875 corresponded to biological processes, 42,566 to cellular components and 61,468 to molecular functions. As expected for plant leaf tissue, most frequent transcript annotations referred to major cellular processes, activities and compartments. Interestingly, a significant number of transcripts obtained annotations to “symplast” (cellular component branch), response to stimulus (biological process branch) and “antioxidant activity” (molecular function branch) (Fig. S6). Multiple GO terms assigned to each transcript resulted in the assignment of each transcript to multiple processes. Enzyme code annotation of the transcripts was performed in Blast2GO. In total, 13,085 transcripts were assigned to specific enzyme code and enzymes hydrolases (5167) were the most prevalent groups present in the gerbera leaf transcriptome followed by transferases 3956), oxidoreductases 2334), lyases (647), ligases (513), and isomerases (468) (Fig. S7).

### Differential gene expression

**Fig. 2:**
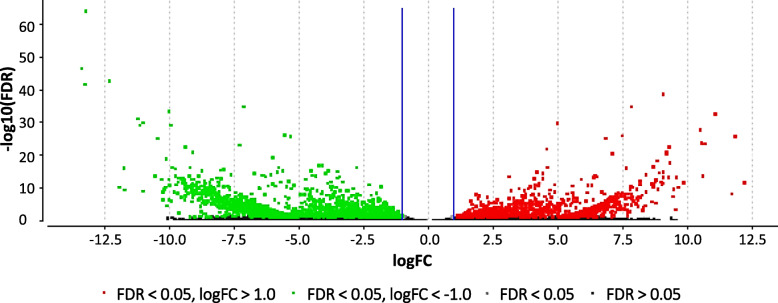
Volcano plot showing transcripts differentially expressed in gerbera breeding lines UFGE 4033 and 06–245-03. UFGE 4033 and 06–245-03 are resistant and susceptible to powdery mildew disease, respectively

**Fig. 3:**
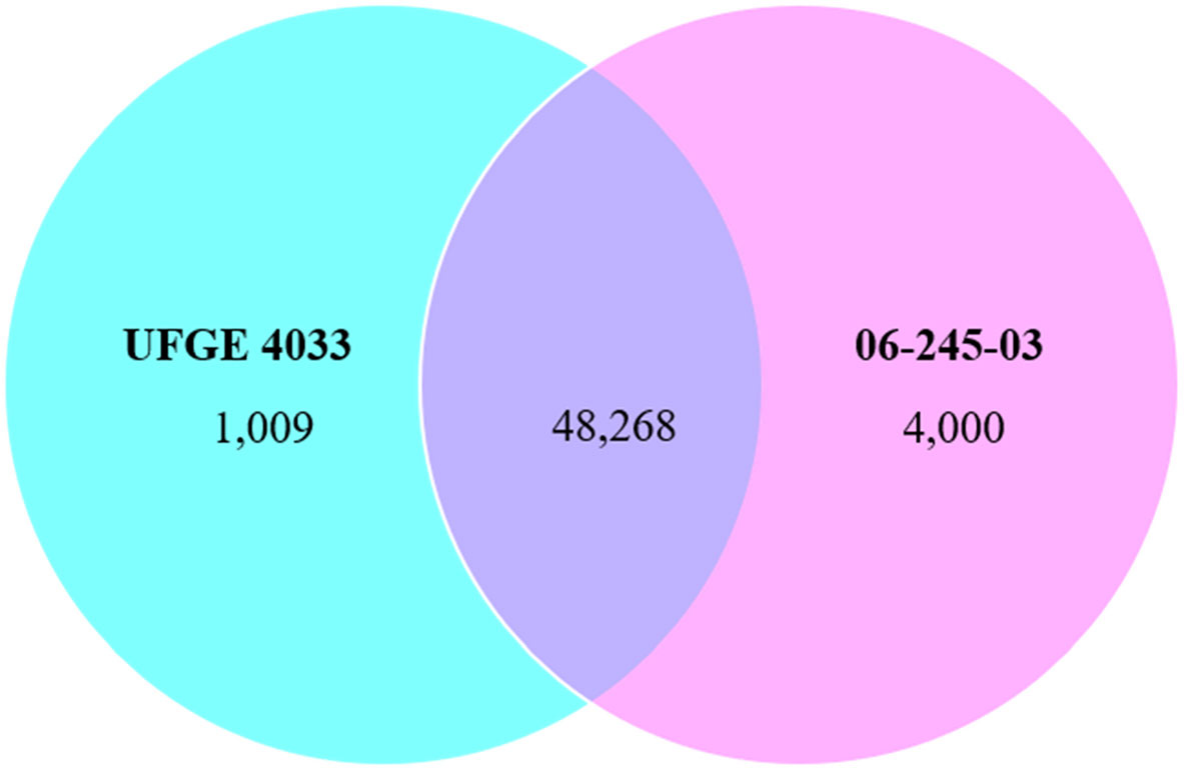
Venn diagram showing transcripts distribution in powdery mildew-resistant (UFGE 4033) and -susceptible (06–245-03) gerbera lines

In total, 398,015,666 PE reads were aligned, and 227,913,186 (57.07%) PE reads successfully mapped to 145,348 transcripts with an average mapping quality of 35.72 (Table S3). The average duplication rate was 56.47% and the average GC content of the mapped reads was 46.51%. These mapped reads from each sample were counted using RSEM and normalized and analyzed for differential expression using Blast2GO. All 145,348 assembled transcripts were submitted to Blast2GO analysis and 130,858 transcripts passed the Blast2GO filtering process (Fig. S2). Of 130,858 transcripts, Blast2GO performed differential expression (DE) analysis on 63,373 that contained ORFs. There were 3213 differentially expressed transcripts (FDR < 0.05) between the resistant and the susceptible gerbera lines. Among the DE transcripts 1190 were up-regulated [log_2_ fold change (logFC > 1)] and 2023 were downregulated (logFC < − 1) in the PM resistant gerbera line as compared to the PM susceptible line, respectively (Fig. 2). A list containing all transcripts subjected to differential expression analysis and their expression values are shown in Table S4. The highest upregulation of 12.15 logFC and downregulation of − 13.42 log FC were observed from DE analysis. A total of 48,268 transcripts were present in both breeding lines, whereas 1009 and 4000 transcripts were present only in UFGE 4033 and 06–245-03, respectively (Fig. 3). Among the differentially expressed transcripts, GO-terms related to “oxidation-reduction”, “transcription”, “phosphorylation” “DNA integration”, “signal transduction” and “transmembrane transport” were identified to be most represented (Fig. S8).

### Transcripts differentially expressed and involved in disease resistance

**Fig. 4:**
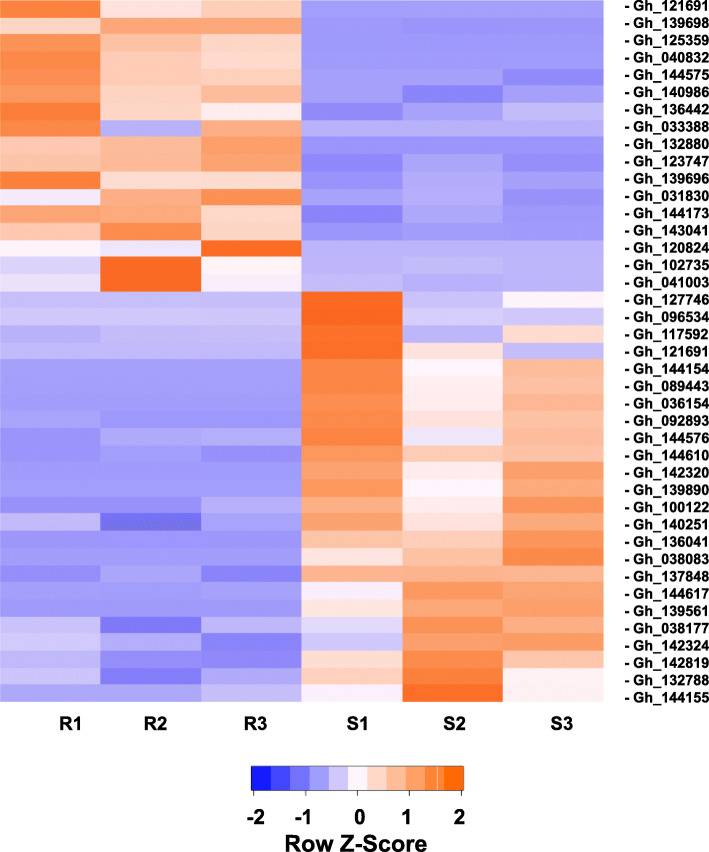
Heat map of relative expression level (FPKM values) of 17 upregulated and 24 downregulated transcripts. These transcripts were functionally annotated to confer disease resistance based on BLAST analysis in powdery mildew resistant (UFGE 4033) and susceptible (06–245-03) gerbera breeding lines. Samples R1-R3 and S1-S3 were three biological replicates of UFGE 4033 (R) and 06-245-03 (S) lines, respectively

Transcripts involved in disease resistance (DR) were selected from the differentially expressed gene list following the functional annotation using the keyword term “disease resistance”, resulting in a selection of 494 transcripts (Table S5). Seventeen and 24 DR transcripts were upregulated and downregulated in the PM-R line UFGE 4033, respectively (Fig. 4, Table 2). Four upregulated DR transcripts, Gh_033388, Gh_120824, Gh_125359 and Gh_132880, had no expression in PM-S line 06–245-03. Two upregulated DR transcripts had a low level of expression (FPKM < 1.0) in the PM-S line and a much higher level of expression (FPKM 9.18 and 12.79) in the PM-R line (Table 2). Eight transcripts, Gh_036154 (logFC = − 6.87), Gh_038083 (logFC = − 4.81), Gh_089443 (logFC = − 5.63), Gh_121691 (logFC = − 7.13), Gh_136041 (logFC = − 5.09), Gh_139561 (logFC = − 6.88), Gh_139809 (logFC = − 5.80), Gh_144154 (logFC = − 8.25), were not expressed in the PM-R line but were expressed in the PM-S line (Table 2). Seven transcripts showed low expression in the resistant line (FPKM < 1.0) but much higher expression in the susceptible line (FPKM from 4.99 to 102.13) (Table 2). Among the upregulated DR transcripts, eight were most similar to *At3g14460*, an *Arabidopsis* gene playing an important role in resistance to fungal diseases [29]. Two transcripts were similar to *At4g27190* and *RPP13*, both involved in disease resistance, and seven transcripts were most similar to *At1g61180*, *At4g27220*, *RGA2*, *RGA3*, or *RML1A* (Table 2). Among the downregulated transcripts, there were nine transcripts most similar to *At3g14460*, two transcripts were similar to *At4g27220*, four transcripts were similar to *At4g27190*, two transcripts were related to leaf rust 10 disease-resistance receptor-like protein kinase-like 1.2, one transcript similar to *RGA1*, *RGA3*, *RPP13*, *RMLA1* and *RGC22* each (Table 2). The highest logFC of the upregulated DR transcripts was 6.08 (Gh_040832 and Gh_139712) whereas among the downregulated was of Gh_142320 with − 8.99 (Table 2).

**Table 2:** Disease resistance transcripts differentially expressed in powdery mildew-resistant (UFGE4033) and susceptible (06–245-03) gerbera breeding lines

Name	logFC	logCPM	*P*-Value	FDR
Gh_040832	6.085	2.277	4.60E-07	2.38E-05
Gh_139712	6.078	2.273	1.45E-06	6.54E-05
Gh_033388	5.805	9.673	0.006639	0.048872
Gh_120824	5.535	1.925	0.003898	0.045307
Gh_125359	5.006	1.624	5.22E-04	0.00953
Gh_132880	4.894	1.572	9.77E-04	0.015498
Gh_041003	3.928	5.419	3.18E-04	0.006445
Gh_139698	3.761	3.100	2.86E-08	2.04E-06
Gh_143041	3.725	2.663	6.74E-06	2.51E-04
Gh_102735	3.723	3.366	0.001046	0.016224
Gh_031830	2.477	11.821	2.61E-04	0.004324
Gh_123747	2.135	2.850	8.19E-05	0.002088
Gh_140986	2.133	2.494	8.40E-04	0.014068
Gh_144575	1.756	2.751	0.001036	0.0161
Gh_144173	1.608	3.456	3.63E-04	0.007158
Gh_139696	1.545	3.335	0.00118	0.017864
Gh_136442	1.174	5.241	0.004415	0.049861
Gh_038177	−1.120	3.805	0.003616	0.043319
Gh_142324	−1.513	3.675	0.002462	0.031527
Gh_132788	−1.882	3.058	4.60E-04	0.008694
Gh_137848	−1.948	5.755	1.33E-08	1.03E-06
Gh_140251	−2.045	10.030	1.46E-05	4.92E-04
Gh_142819	−2.079	2.640	3.42E-04	0.006811
Gh_144576	−3.448	6.374	1.95E-05	6.34E-04
Gh_117592	−3.499	3.528	0.003077	0.038091
Gh_144155	−3.837	1.906	0.002783	0.034983
Gh_100122	−4.221	3.130	8.24E-08	5.17E-06
Gh_038083	−4.811	1.434	0.001955	0.026137
Gh_092893	−4.903	2.617	8.35E-07	4.04E-05
Gh_136041	−5.091	1.572	4.89E-04	0.009057
Gh_144610	−5.528	1.815	3.30E-05	9.85E-04
Gh_089443	−5.631	1.873	1.03E-04	0.002544
Gh_127746	−5.781	4.496	3.81E-04	0.007446
Gh_139809	−5.797	1.977	4.31E-05	0.001222
Gh_036154	−6.867	2.749	4.37E-08	2.97E-06
Gh_139561	−6.882	2.763	5.21E-10	5.59E-08
Gh_144617	−7.047	2.899	3.50E-09	3.06E-07
Gh_121691	−7.130	2.954	0.002735	0.034501
Gh_096534	−8.125	5.553	0.002083	0.027624
Gh_144154	−8.248	3.948	4.84E-12	8.29E-10
Gh_142320	−8.994	4.651	5.11E-17	2.35E-14

logFC is the log_2_ fold change of the expression value

logCPM are the log counts per million reads

FDR false discovery rate. Transcripts were considered differentially expressed based on FDR < 0.05

### Quantitative RT-PCR (RT-qPCR) validation of differentially expressed *R*-genes

**Fig. 5:**
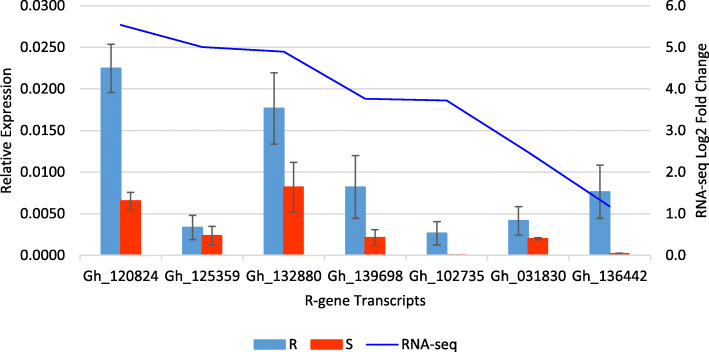
Quantitative RT-PCR validation of seven differentially expressed *R*-gene transcripts from gerbera transcriptome analysis. These transcripts were differentially upregulated in powdery mildew resistant (R) line (UFGE 4033) versus powdery mildew-susceptible (S) line (06–245-03). Expression of transcripts were normalized to the endogenous control *Gerbera hybrida* actin gene and the relative expression was calculated using 2{−Ct (gene of interest)-Ct (actin)}. The blue line shows the Log2 fold change expression of the transcripts from RNA-seq data. Error bars represent standard errors

In order to validate the expression of the *R*-genes identified from the RNA-seq analysis, we randomly selected seven upregulated *R*-genes and performed RT-qPCR. RNA-seq analysis results indicated that these genes were differentially upregulated in the resistant line as compared to the susceptible line, and Gh_120824, Gh_125359, Gh_132880, Gh_139698, Gh_102735, Gh_031830 and Gh_136442 had the logFC values of 5.53, 5.01, 4.89, 3.76, 3.72, 2.47, and 1.17, respectively. Our RT-qPCR data showed similar higher expression levels for Gh_120824, Gh_132880, Gh_139698, Gh_031830 and Gh_102735 (Fig. 5). The expression levels for Gh_102735 and Gh_136442 were almost negligible in the susceptible line but high in the resistant line, thus the upregulation of this *R*-gene transcript seemed to be more pronounced in RT-qPCR than in RNA-seq analysis. One of seven *R*-gene transcripts, Gh_125359, showed a logFC of 5.01 in RNA-seq data but had similar expression levels between the resistant and the susceptible line in the RT-qPCR analysis.

### Putative susceptibility (*Mildew Locus O*) genes in the gerbera transcriptome

In total, 15 putative disease susceptibility (*S*) transcripts belonging to the *MLO* gene families were identified in the gerbera transcriptome based on annotation with the Blast2GO database (Table S6). Four of the S transcripts were similar to *MLO* and lacked expression or were downregulated (< − 1.0) in the resistant line. Gh_037931, Gh_124463, Gh_124934, and Gh_125664 were similar to *mlo*-like protein 8, *MLO*-like protein 6, *MLO*-like protein 12 and *MLO*-like protein 11, respectively (Table 3). Three transcripts (Gh_035301, Gh_116552, Gh_141139) were similar to *MLO* and their expression levels appeared to be higher in the resistant line than in the susceptible line (Table S6), buttheir expression levels were not significantly different between the resistant and susceptible line (FDR < 0.05) (Table 3).

**Table 3:** *Mildew Locus O* (*MLO*)-like transcripts identified in the gerbera leaf transcriptome. The listed *MLO* transcripts were absent or less expressed in the powdery mildew-resistant (UFGE 4033) gerbera line but were only present or highly expressed in the susceptible (06–245-03) breeding line

Name	logFC	Functionality	Length (bases)
Gh_037931	−4.00382	mlo-like protein 8	262
Gh_124463	−1.0435	MLO-like protein 6	1989
Gh_124934	−1.48785	MLO-like protein 12	1849
Gh_125664	−1.17083	MLO-like protein 11	2992

Log_2_fold change was determined by differential expression analysis. Transcripts were considered differentially expressed based on FDR < 0.05. Functionality was determined using Blast2GO

### SSRs and SNPs

**Fig. 6:**
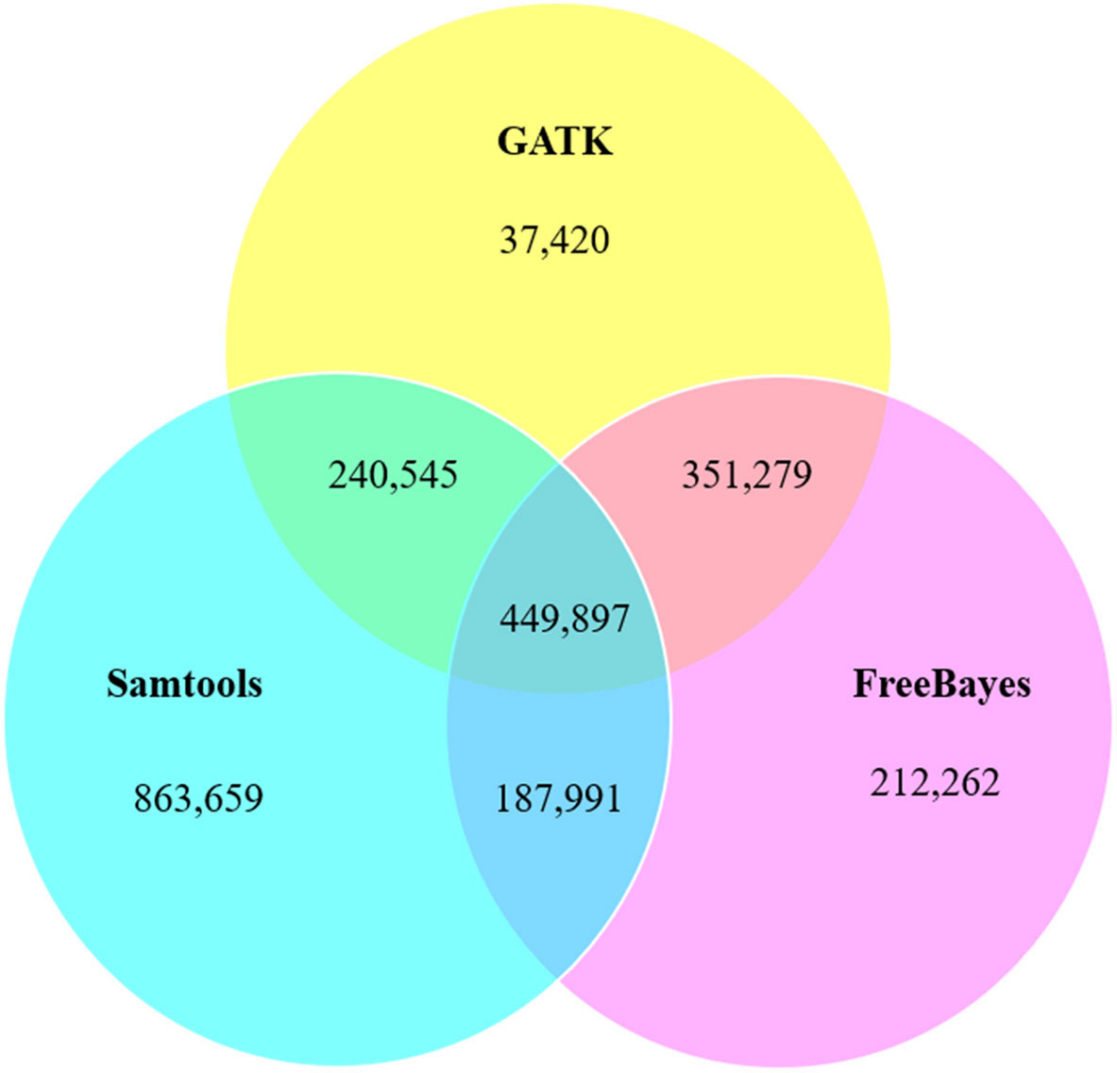
Venn diagram showing the distribution of SNPs in gerbera. SNPs were identified by GATK, Samtools and FreeBayes pipelines between powdery mildew resistant (UFGE 4033) and susceptible (06–245-03) gerbera breeding lines

There were 1,742,092, 1,201,429 and 1,079,141 SNPs identified by GATK v1.7, SAMtools v2016.0.109 and FreeBayes v1.0.2–6, respectively (Fig. 6; Table S7). A total of 449,897 SNPs was identified by all three pipelines. SAMtools discovered the highest number of SNPs and 863,659 SNPs that were not identified by the other two pipelines. GATK discovered the lowest number of SNPs and 37,420 SNPs that were not discovered by the other two pipelines. FreeBayes detected 212,262 SNPs that were not discovered by the other two pipelines (Fig. 6).

**Fig. 7:**
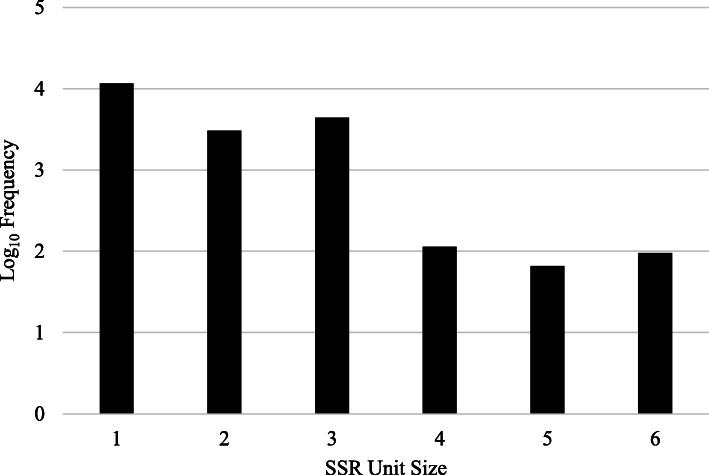
Distribution of simple sequence repeats (SSRs) with different motif unit sizes in gerbera transcriptome. SSRs with one – six motif sizes were identified using Misa tools. Bars represent the frequencies (log_10_) of SSRs containing various lengths of nucleotide repeats

Of the 145,348 transcripts analyzed with the MISA program, 14,864 transcripts contained 19,393 SSRs (Table S8). There were 3235 transcripts each containing more than one SSR, and 2235 SSRs were present in compound formation. There were 11,652 mononucleotide SSRs, 3053 dinucleotide SSRs, 4413 trinucleotide SSRs, 114 tetranucleotide SSRs, 66 pentanucleotide SSRs and 95 hexanucleotide SSRs identified in the gerbera transcriptome (Fig. 7; Table 4).

**Table 4:** Distribution of simple sequence repeats (SSRs) with different motif sizes identified in gerbera leaf transcriptome

SSR type	Number of SSRs
Mononucleotide repeats	11,652
Dinucleotide repeats	3053
Trinucleotide repeats	4413
Tetranucleotide repeats	114
Pentanucleotide repeats	66
Hexanucleotide repeats	95

## Discussion

RNA-sequencing has been widely used to understand gene expression, discover DE transcripts and develop molecular markers in plants. RNA-seq is independent of pre-existing databases of expressed genes and therefore can be used to construct an unbiased profile of gerbera gene expression in relation to PM. Previously RNA-seq has been used in gerbera to profile transcriptome changes in response to physiological disorders and biotic and abiotic stresses, including stem bending, *Botrytis* infection, and cold stresses [23, 27, 30]. While the PM resistance has been transferred to new cultivars developed by the University of Florida using the resistance source UFGE 4033, the cultivar development process relied on classical breeding and PM screening. Identification of genes conferring resistance to PM can speed up the development of PM-resistant cultivars using marker-assisted breeding and novel methods of gene transfer [31]. In this study, transcriptome sequencing was used to profile the leaf transcriptome of two gerbera breeding lines, UFGE 4033 and 06–245-03, that differed in resistance to powdery mildew and to analyze gene expression specifically during the post-inoculation stage when most of the DR genes are expressed in the breeding lines due to exposure to PM.

Gerbera transcripts were most similar to those of *H. annuus* and *C. cardunculus* var. *scolymus*, both of which belong to the Asteraceae, as does gerbera. PM has been a challenging problem in both *H. annuus* and *C. cardunculus* var. *scolymus*. Studies have been performed in *H. annuus* to investigate PM resistance and host-pathogen interactions [32–35].

In total, 145,348 transcripts were identified from the transcriptome assembly. More than 57% of reads mapped to the transcriptome which could be attributed to multi-mapping reads onto exons shared by different transcripts of the same gene and the reads coming from unannotated transcripts remain unmapped [36]. In human, 70–90% of reads are expected to map to a reference genome whereas lower mapping rates are observed while mapping to transcriptome [36, 37]. Differential expression analysis between UFGE 4033 and 06–245-03 identified 3213 transcripts that were differentially expressed, of which 1190 were upregulated and 2023 were downregulated in UFGE 4033. In the DEGs, GO terms related to oxidation-reduction GO:0055114, protein phosphorylation GO:0006468, transmembrane transport GO:0055085 and signal transduction GO:0007165 were enriched. Oxidation-reduction enzymes take part in production of reactive oxygen species (ROS) and elevate the host resistance to pathogens. ROS are able to restrict the pathogens by killing them or act as a signaling molecule to synthesize antimicrobial compounds and has been studied in fungal disease resistance [38]. Enrichment of GO:00055114 occurred during the activation of apple resistance to *Botryosphaeria dothidea*, which causes canker [39]. Transmembrane transport and phosphorylation of transcription factors have been reported to regulate plant defense and fungal resistance in Arabidopsis [40]. Signal transduction mediated host resistance is one of the common resistance mechanisms against biotrophic fungal pathogens [41].

This study identified 41 transcripts that were differentially expressed and involved in “disease resistance”. Thirty-six of the DEGs contained the NB-ARC domain and could be classified as *R*-gene transcripts. The NB-ARC domain plays a very important role in the largest class of *R*-genes, the nucleotide-binding site (NBS) and leucine-rich repeat (LRR) (NBS-LRR) genes [42]. This class of *R*-genes confers resistance to bacterial, fungal and viral plant pathogens as well as to nematodes and aphids [43, 44]. NBS-LRR genes encode for proteins that mainly function as intracellular receptors to perceive the effector from pathogens. A number of *R*-genes can confer PM resistance in plants [11, 45, 46]. For example, in *Medicago truncatula*, Foster-Hartnett et al. identified genes involved in hypersensitive response (HR) and basal defense for *Erysiphe pisi* [47]. In the present study, transcripts related to GO-terms: GO55114 and GO45454 were found to be involved in oxidation-reduction process. Oxidation-reduction process during plant defense to microorganisms is known to contribute to production of reactive oxygen species (ROS) and regulate HR [48, 49]. In wheat and barley, NBS-LRR encoding genes, *Pm* and *Mla*, confer PM resistance to *Blumeria graminis* [50]. Similarly, genes present at the *Run* and *Ren* loci contain the NBS-LRR domain causing PM resistance to *Uncinula necator* in grapevine [51]. In the present study, differentially expressed gerbera “disease resistant” transcripts are highly similar to some of the previously identified *R*-genes like *RGA, RPP13, RML1A*, At3g14460, At4g27190 and At4g27220.

Among the 36 gerbera NBS-LRR transcripts, eight were functionally similar to At3g14460. Based on GO biological processes classification, At3g14460 protein is activated in the nucleus and functionally involved in defense response to fungi by binding ADP, ATP and adenylate cyclase activity. There were two genes similar to At4g27190. There are five homologs of At4g27190 reported in *O. sativa* and one in *P. trichocarpa*. This is also involved in pathogen recognition and defense response and belongs to the NB-LRR family. There were two genes identified that were similar to *RPP13*, which confers resistance to *Hyaloperonospora parasitica*, an oomycete that causes downy mildew in a wide range of plants, including Arabidopsis, Brassicas and Cucurbits [52, 53]. *RPP13*-like transcripts were also found to be upregulated in downy mildew resistant New Guinea impatiens compared to garden impatiens susceptible to the disease [54]. RPP13 is localized in the plasma membrane and cytoplasm and confers defense response resulting in incompatible interaction and hypersensitive response [52]. There was one gene similar to *RGA2*, which confer broad resistance to blight in *Solanum bulbocastanum* caused by all races of *Phytophthora infestans.* While *RGA2* confers horizontal resistance, there was a gene similar to *RGA3*, which if present in susceptible haplotype, act as pseudogenes created by deletions and mutations. There were two *RLM1A*-like transcripts differentially expressed in gerbera, with one expressed only in UFGE 4033. Gene *RLM1* confers resistance to a hemibiotrophic fungus, *Leptosphaeria maculans*, causing black leg in Brassica crops [55]. Despite displaying a necrotrophic reaction, *L. maculans* similar to many biotrophic pathosystems, has established a gene-for-gene relationship with both *Brassica napus* and *A. thaliana* by signal transduction [55, 56]. There were two genes similar to At1g61180 and At4g27220, respectively. Both genes belong to NB-LRR family and are involved in disease resistance, however, little is known about these genes.

Disease resistance protein At4g27190 belongs to the NB-LRR family [41]. Chen et al. reported that two At4g27190-like proteins significantly expressed in woodland strawberry (*Fragaria vesca*) during infection by *Phytophthora cactorum* [57]*.* Not all disease-resistant transcripts identified were upregulated in gerbera. There were nine transcripts similar to At3g14460, two genes similar to At4g27220, four genes similar to At4g27190, two genes related to leaf rust 10 disease-resistance receptor-like protein kinase-like 1.2, one gene similar to *RGA1, RGA3, RPP13, RML1A* and *RGC22* each, and one gene involved in grave disease carrier protein were identified to be downregulated in the given samples. Further study is required to understand the cause of these transcripts to be downregulated. The presence of this diversity of disease resistance transcripts in both upregulated and downregulated groups indicates the functional diversity among the transcripts to defend PM pathogen.

*Mildew locus O* genes encode a plasma membrane protein with seven trans-membrane helices and a C-terminal calmodulin-binding domain acting as a prerequisite for successful colonization of PM pathogens in numerous crops [58–61]. Previously, PM susceptibility due to *MLO* gene families by facilitating fungal penetration phase into the host has been reported for PM pathogens in numerous crops including ornamentals like petunia and rose and when mutated deployed broad-spectrum, durable resistance to PM in these crops [62, 63]. In this study, we identified four MLO-like transcripts however, these transcripts were not differentially expressed. These *GhMLO* genes in the gerbera genome could assist in the fungal penetration although further studies are warranted to confirm this hypothesis. Availability of reference gerbera genome and full gene lengths of *MLO* genes will help in this hypothesis validation in the future.

Identification of SNPs and SSRs from transcriptome sequences can be used in construction of genetic linkage maps, high-resolution gene and genome mapping, and marker-assisted breeding. This study has identified 1,724,092 SNPs using Samtools, 1,079,141 SNPs using GATK and 1,201,429 SNPs using FreeBayes pipelines and 19,393 SSRs using MISA. These large numbers of SNPs indicate a high level of heterozygosity between the PM-resistant and susceptible gerbera breeding lines. Previously, Gong and Deng identified 893 EST-SSRs from 16,998 ESTs in gerbera using in silico analysis [64]. Besides this, there are no known efforts recently to identify SSR markers using next generation sequencing. The accuracy of the SNPs identified from this study could be obtained by comparing genotyping data developed using various platforms like genotyping by sequencing. However, the SNPs in the intronic region of gerbera genome is not present in this study. Therefore, intronic SNPs developed from other methods cannot be compared with the SNPs identified from this study. SNPs from the candidate transcripts putatively involved in PM resistance can be identified and validated to develop potential markers for PM resistance in gerbera.

In summary, we performed extensive analysis and characterization of the leaf transcriptomes of two gerbera daisy genotypes exhibiting strong resistance or extreme susceptibility to PM, obtained 145,348 contigs, and functionally annotated 46,258 transcripts. For the first time, this study revealed genome-wide gene expression differences in gerbera in response to PM. We identified 36 disease-resistance gene transcripts belonging to *R*-gene families. These transcripts are highly valuable genomic resources for developing molecular markers to aid rapid screening of breeding populations for PMR and development of multiple series of new PMR cultivars. These transcripts can serve as strong candidate genes for genetic mapping and molecular cloning of *R*-gene(s) controlling the strong resistance in UFGE 4033. Additionally, we identified *MLO*-like transcripts that were expressed or highly expressed only in the PM-susceptible genotype. These transcripts will enable a more thorough understanding of the roles of *MLO* genes in gerbera and they can serve as strong susceptibility gene candidate for gene editing and knocking down for improving gerbera resistance to PM. The SSRs and SNPs identified in this study provide a vast pool of sequence polymorphisms for developing molecular markers that can be used in studying gerbera diversity and genetic fidelity and implementing marker-assisted selection in cultivar development. Genomic resources in gerbera are meager as compared to other globally popular ornamental crops in comparable demand as gerbera. At the current situation, this study will add to the genomic resources for gerbera research. Although obtaining the full lengths of the transcripts is not trivial using RNA-seq approach, the availability of reference genome in the future will facilitate the goal of extracting complete gene sequences conferring traits of interest.

## Conclusions

Powdery mildew resistance is a very important trait in gerbera, which is one of the top five most important floricultural crops in the world. Sequencing, *de-novo* assembly and comprehensive analyses of the leaf transcriptomes of a pair of gerbera breeding line with contrasting phenotypes in powdery mildew resistance led to the identification of 41 disease resistance gene-like transcripts that were differentially expressed in the resistant gerbera line. This study revealed four *Mlo-*like transcripts only or highly expressed in the powdery mildew susceptible gerbera line. These results represent the first report of candidate resistance genes and susceptibility genes in gerbera, providing highly valuable targets for development of molecular markers for and isolation of the gene(s) controlling the powdery mildew resistance and facilitating the use of marker-assisted selection and gene editing technologies in gerbera. This study also identified a large number of SNPs and SSRs that can be used to develop genome-wide genetic linkage maps, locate horticulturally important genes, and study genetic diversity in gerbera, whose genomic resources have been very scarce in the public domain.

## Methods

### Plant materials

Six gerbera breeding lines with excellent horticultural traits, from the University of Florida gerbera germplasm collection, were screened to find the most PM-susceptible line for use in this study. To select the most susceptible line, these selections were grown from crowns in four-inch plastic pots using a soilless potting mixture Faffard® 3B (50% Canadian peat and 50% of the mixture of vermiculite, pine bark and perlite) (Agawam, MA, USA) and kept in the mist chamber in a greenhouse at the Gulf Coast Research and Education Center, Wimauma, FL, USA. The mist chamber was set to run once every 30 min for one minute during the day. After two weeks, when plants were established, they were moved out of the mist chamber. Two weeks later, plants were transplanted to seven-inch plastic pots and grown for a month. The growing plants were then screened for PM susceptibility.

Gerbera line UFGE 4033, developed by the University of Florida gerbera breeding program, was used as a resistant control and grown along with the susceptible lines. UFGE 4033 was developed from a cross between the PM-resistant line UFGE 31–19 and a susceptible line UFGE 35–4. Hence, UFGE 4033 was a hybrid and was expected to be heterozygous [17]. The PM resistance trait in UFGE 4033 had been transferred into a number of cultivars [18]. Therefore, this plant was used as a resistant line for transcriptome sequencing.

Six susceptible lines, along with the resistant control, UFGE 4033, were randomly distributed in three blocks using a randomized complete block design. These plants were naturally infected with PM, which was widespread in the greenhouse and other controlled structures at the facility. The disease was allowed to develop for one month and then rated once every week for five weeks using a rating scale of 1 to 10, where 1 = no disease, 2 = trace to 10%, 3 = 10 to 20%, 4 = 20 to 30%, 5 = 30 to 40%, 6 = 40 to 50%, 7 = 50 to 60%, 8 = 60 to 70%, 9 = 70 to 80%, and 10 = 80 to 100% of leaf surfaces covered with PM [65]. The most susceptible line from the experiment was used in transcriptome analysis.

### RNA isolation and library preparation

Gerbera breeding line UFGE 4033 and 06–245-03 were selected for transcriptome sequencing. UFGE 4033 and 06–245-03 were clonally propagated as described above to produce three individuals, which were used as three biological replicates. These individuals were grown in a greenhouse at the daytime temperature of 25 °C – 32 °C and the nighttime temperature of 18 °C – 22 °C in nearly 12 h daylight period. The plants were fertigated weekly with 250 ppm of 15–16-17 NPK (Cat. # G99210, Peters Professional, Dublin, Ohio). No fungicides were sprayed on the plants during the experimental period. When the clonally propagated plants were thirty days old, they were inoculated by dusting the PM spores onto the plant canopy. After three to four days of inoculation, when the first symptoms appeared on 06–245-03, young leaf tissues were collected into 50 mL sterile plastic tubes from the individuals, instantly frozen in liquid nitrogen and transferred to − 80 °C. The leaf samples were shipped on dry ice to Novogene Co. in Beijing, China for RNA extraction and sequencing.

Total RNA was extracted from the young leaf tissues using the TRIzol method [66]. The concentration of total RNA was determined on a Nanodrop spectrophotometer (Thermo Scientific, Waltham, MA, USA); RNA degradation, potential contamination and purity was assessed on 1.0% agarose gel (0.5× TBE solution; 180 V voltage for 16 min); RNA quantification and integrity was determined on an Agilent 2100 bioanalyzer (Santa Clara, CA, USA). RNA preparations with high quality were used for library preparation and sequencing.

Library construction was performed using the NEB Next® Ultra RNA Library Prep Kit (New England Biolabs, Ipswich, MA, USA). Messenger RNAs (mRNA) were enriched from total RNA using oligo (dT) beads. Enriched mRNAs were fragmented randomly in fragmentation buffer using the Elute Prime Fragment Mix from Illumina TruSeq™ RNA sample prep kit v2 (Illumina, San Diego, CA, USA). cDNA was synthesized from fragmented mRNA using random hexamers and reverse transcriptase using Illumina TruSeq™ RNA sample prep kit v2 (Illumina, San Diego, CA, USA) using the developer’s protocol. After first-strand synthesis, a custom second-strand synthesis buffer (Illumina, San Diego, CA, USA) was added with dNTPs, RNase H and *Escherichia coli* polymerase I to synthesize the complementary strand by nick-translation to form double-stranded stable cDNA. AMPure XP beads were used to purify the synthesized cDNA. cDNA was further purified, terminal ends were repaired, poly-A tails were added to prepare the final cDNA. Sequencing adapters were then ligated to the final cDNA for unique identification. These adapters added cDNAs were then size selected and enriched by polymerase chain reaction (PCR) to create the library of cDNAs to be sequenced. The selected cDNA fragments size of 250 ~ 300 bp insert in the cDNA library (non-directional). Quality of the library was assessed by measuring the concentration using a Qubit 2.0 fluorometer (Life Technologies, Carlsbad, CA, USA), adjusting the library concentration to 1 ng/μl, verifying the insert size of the amplicon using an Agilent 2100 (Bioanalyzer) and finally quantifying to higher precision by quantitative PCR (qPCR) (library activity > 2 nM). The quality-controlled library was then used for sequencing.

### Transcriptome sequencing, quality control and filtering

Sequencing of the libraries was performed on an Illumina HiSeq 2000 platform using two lanes of an Illumina flowcell. Original raw data from HiSeq 2000 were converted to sequence reads by base calling and stored as fastq file containing sequences and corresponding sequencing quality description. FASTQC v0.11.1 pipelines were used to perform quality analysis, removing adapters and filtering the low-quality sequences. Adapters (RNA 5′ Adapter (RA5): 5′-AATGATACGGCGACCACCGAGATCTACACTCTTTCCCTACACGACGCTCTTCCGA TCT-3′ and RNA 3′ Adapter (RA3): 5′-GATCGGAAGAGCACACGTCTGAACTCCAGTCAC (6-nucleotide index) ATCTCGTATGCCGTCTTCTGCTTG-3′) ligated to the sequences prior to sequencing were removed from the sequenced reads and paired-end reads of 150 bp excluding the adapters were generated. Sequence description was used to assess the quality of sequenced data. Sequencing error rate for each base was assessed using Phred score. Distribution of GC content was also evaluated to detect AT/GC separation in the sequence. After quality assessment, sequence reads were subjected to different filters. Reads containing adaptor sequences were discarded. Sequenced reads containing more than 10% of uncertain nucleotides (*N* > 10%) were also discarded. Based on Phred scores, if the sequenced reads contained more than 50% of nucleotides with Phred scores below 20 then those reads were also eliminated. After filtering, sequenced reads containing less than 10% of unknown nucleotides and Phred scores ≥20 were retained as clean reads. The quality of the clean reads was assessed by FASTQC method and used for de novo assembly construction and downstream analysis.

### *De novo* assembly

The *de novo* assembly pipeline developed by the National Center for Genome Analysis Support (NCGAS) at Indiana University (https://github.com/NCGAS/de-novo-transcriptome-assembly-pipeline) was used to create an assembly from the cleaned paired-end reads of both UFGE 4033 and 06–245-03. This pipeline uses four different assemblers: Trinity, TransAbyss, Velvet and SoapDenovo [67–70]. Trinity (trinityrnaseq/2.4.0) was run using default parameters and normalization at 50x coverage (Trinity --normalize_max_read_cov --seqType fq --max_memory 200G --left reads_1.fq.gz --right reads_2.fq.gz --CPU 6) [67]. TransAbyss (transabyss/2.0.1) was run with k-mers of k35, k45, k55, k65, k75 and k85 and the following command (transabyss -k 35 --pe $reads1 $reads2 --outdir $OD --name k35.transabyss.fa --threads 4 -c 12) [68]. K-mers 35, 45 and 55 were used in one command and k-mers 65, 75 and 85 in a second command. The outputs from each command were then combined. Velvet (velvet/1.2.10) was used with k-mers of 35, 45, 55, 65, 75 and 85 (velveth oases.35 35 -shortPaired -fastq -separate $left $right and) and insert size of 260 (velvetg oases.35 -read_trkg yes -ins_length 260 and). The transcripts generated from different k-mers (35, 45, 55, 65, 75, and 85) were combined by the program (sed -i ‘s/>/>Velvet.k35./g’ oases.35/transcripts.fa. Soapdenovo (soapdenovotrans/1.03) was run with k-mers of 35, 45, 55, 65, 75 and 85 (SOAPdenovo-Trans-127mer all -s config_file -K 35 -o output35 and). Transcripts assembled by the above four assemblers were merged, retaining the name of assemblers generating each contig, and BLAST was performed using Evigene [71] to retain the high quality contigs. The contigs that were generated by individual assemblers but not included in the final merged assembly were retained but analyzed separately.

### Functional annotation

Functional annotation of the assembled contigs was done using Blast2GO version 5 [72]. The gerbera contigs were compared to the NR database using BLASTp at a cutoff value of 1.0E-03 and the 20 top hits with the highest similarity were retained. Top hits obtained from the BLAST searches were then submitted to the Gene Ontology (GO) database to obtain the GO terms and GO ID for the hits from BLASTx searches. Annotation of the contigs with the assigned GO terms was performed by using the E-value-Hit-filter of 1.0E-06 with annotation cutoff of 55 and GO weight of 5 and other default parameters of Blast2GO [73]. Transcripts with assigned GO terms were submitted to the Web Gene Ontology Annotation Plot (WEGO) 2.0 [28] for categorization of GO terms in the three main GO categories.

### Quantification

Quantification of the reads was performed by counting the mapped raw reads to the transcriptome assembly. Bowtie2 was used to perform un-gapped mapping using default parameters and RSEM was used to quantify the transcript expression levels [74, 75]. Quality of read mapping was assessed using Qualimap and Samtools pipelines [76, 77]. RSEM uses expectation maximization to infer transcript level expressions. The sequence reads that mapped to each transcript were counted, normalized and calculated as fragments per kilobase of transcript (FPKM) values.

### Differential gene expression analysis

Pairwise comparisons of UFGE 4033 and 06–245-03 reads were performed by grouping the reads from the three biological replicates of 06–245-03 as a susceptible group and from the three biological replicates of UFGE 4033 as a resistant group. Differential expression was performed in Blast2GO using the builtin pipeline of the software. Trimmed mean of M value (TMM) normalization was performed and the expression analysis was performed using disease resistance and susceptibility as experimental factors and three susceptible and three resistant samples were assigned as reference and contrast conditions, respectively. Exact test was performed to determine the level of significance. Transcripts were considered differentially expressed using the FDR < 0.05. The log2fold change of each transcript was noted and used in further analysis. The log2fold change of > 1.0 was used to call upregulated transcripts and logFC < − 1 was used to call downregulated transcripts. Transcripts with expression level between 1 to − 1 were not considered to be differentially regulated.

### Quantitative RT-PCR analysis

Total RNA was extracted from the leaf tissues collected for RNA-seq (described above) using three biological replicates of the resistant and the susceptible line using the RNeasy plus mini kit (Cat. # 74134, Qiagen, Hilden, Germany) using the manufacturer’s protocol. Quality of the extracted RNA was assessed using a nanodrop spectrophotometer. Reverse transcription was performed to convert 500 ng RNA of each sample to cDNA using High-Capacity cDNA Reverse Transcription Kits (Cat. # 4368814, Applied Biosystems, Foster City, CA) using the manufacturer’s chemistry and protocol. Briefly, 2.0 μL of 10x RT buffer, 0.8 μL of 25x dNTP Mix (100 mM), 2.0 μL of 10x RT random primers, 1 μL of MultiScribe™ reverse transcriptase, 4.2 μL of nuclease-free water, and 500 ng of RNA for each sample for cDNA conversion. cDNA synthesis was performed at 25 °C for 10 min, 37 °C for 120 min, and 85 °C for 5 min. RT-qPCR reactions were performed using the chemistry of Applied Biosystems (SYBR green, Cat. # 100029284, Applied Biosystems). The reaction mixture was 20 μL, consisting of 10 μL of SYBR green, 1 μL of forward and reverse primers, 2 μL of cDNA and 7 μL of nuclease-free water. The PCR was performed with 50 °C for 2 min., 95 °C for 3 min., 95 °C for 30 s., 60 °C for 15 s., 95 °C for 15 s. and 60 °C for 30 s. on a QuantStudio 3 realtime PCR machine (Applied Biosystems). Primers used in the RT-qPCR experiment were designed using primer3 software (https://bioinfo.ut.ee/primer3-0.4.0/) and are listed in Table S9. Primers for the actin gene in gerbera was used from Ge et al., and Kuang et al. [27, 78]. The expressions of the target genes were normalized to an endogenous *G. hybrida* actin gene and relative expressions were calculated using 2^{−Ct(gene of interest)-Ct(actin)}^ [79].

### Identification of simple sequence repeats

Identification of SSRs in the transcriptome was performed using the MISA program [80]. SSRs were called based on the number of repetitions of nucleotides. SSRs with mono-, di-, tri-, tetra-, penta- and hexa-nucleotides were identified only if mononucleotides were repeated for at least 10 times, dinucleotides for at least 6 times, tri-, tetra-, penta- and hexanucleotides for at least 5 times. For compound SSRs, a maximum interruption distance between consecutive SSRs was set to be 100 bp. Only SSRs with at least 100-bp flanking sequences on both sides of the repeats were retained.

### Identification of single nucleotide polymorphisms

Identification of SNPs was done using three pipelines: GATK v1.7, SAMtools v2016.0.109 and FreeBayes v1.0.2–6 [77, 81, 82]. Any SNPs with a quality score < 20 were discarded. The SAMtools mpileup pipeline and its default parameters were used to call SNPs. The reference transcriptome was indexed using the faidx command and .bam files were sorted before running the pipeline. GenotypeGVCFs command line was used in GATK with default parameters to call SNPs using this pipeline and using transcriptome as a reference. FreeBayes default pipeline was used to call SNPs in UFGE 4033 and 06–245-03 separately and later jointly analyzed to call consensus SNPs.

## Supplementary Information

**Additional file 1: Table S1.** Description of read data, quality control and GC content of UFGE 4033 and 06–245-03 gerbera breeding lines. **Table S2.** Functional annotation of gerbera transcripts performed using Blast2GO. **Table S3.** Mapping statistics of UFGE 4033 and 06–245-03 samples to de novo transcriptome assembly using Samtools and Qualimap. Samples R1, R2 and R3 represent three biological replicates of UFGE 4033 and S1, S2 and S3 represent three biological replicates of 06–245-03. **Table S4.** List of gerbera transcripts from powdery mildew resistant (UFGE 4033) and susceptible (06–245-03) breeding lines from the differential expression analysis of gerbera transcriptome. **Table S5**. List of gerbera transcripts from powdery mildew resistant (UFGE 4033) and susceptible (06–245-03) breeding lines functionally annotated as disease resistance in gerbera transcriptome. **Table S6.** List of Mildew locus O (*MLO*)-like transcripts present in the gerbera transcriptome constructed using powdery mildew resistant (UFGE 4033) and susceptible (06–245-03) breeding lines. **Table S7.** Single nucleotide polymorphisms identified between powdery mildew resistant (UFGE 4033) and susceptible (06–245-03) gerbera breeding lines using RNA-seq and SAMtools, FreeBayes and GATK pipelines. **Table S8.** Statistics on simple sequence repeats identified in gerbera transcriptome using powdery mildew resistant (UFGE 4033) and susceptible (06–245-03) breeding lines and MISA tools. **Table S9.** Primers for RT-qPCR analysis of differentially up-regulated *R*-genes in gerbera for powdery mildew resistance.

**Additional file 2: Fig. S1.** Gerbera breeding lines 06–245-03 (left) susceptible to powdery mildew (PM) and UFGE 4033 (right), resistant to PM used for RNA-sequencing. **Fig. S2.** Powdery mildew (PM) symptoms in gerbera A) Whole plant infected with PM B) White fungal spores on the adaxial leaf surface C) White PM spores on capitulum D) White PM spores observed on the peduncle and lower flower surface E) PM conidia as observed under a microscope with a 40x objective. **Fig. S3.** Functional annotation analysis of gerbera RNA-seq data of powdery mildew resistance and susceptible breeding lines using Blast2GO. **Fig. S4.** Top-hits species distribution of gerbera transcriptome by comparing gerbera transcripts to the viridiplantae database using Blast2GO. **Fig. S5.** Histogram showing the frequency distribution of gerbera transcripts with which the number of Gene Ontology (GO)-terms are associated. The figure was created using Blast2GO analysis. **Fig. S6.** Annotation of Gene Ontology-terms assigned to the gerbera transcriptome using WEGO2.0 analysis. **Fig. S7.** Frequency distribution of enzyme class distribution of gerbera transcripts using Blast2GO. **Fig. S8.** Frequency distribution of Gene Ontology (GO)-Terms that were enriched among the differentially expressed gerbera transcripts.

## Data Availability

The data charts supporting the results and conclusions are included in the article and additional files. All sequences generated by sequencing for this study can be found in the NCBI Short Reads Archive (SRA) BioProject PRJNA647707 (https://www.ncbi.nlm.nih.gov/sra/?term=PRJNA647707).

## Abbreviations

PM: Powdery mildew
RNA-seq: RNA sequencing
SNP: Single nucleotide polymorphism
SSR: Simple sequence repeat
GATK: Genome analysis toolkit
FDR: False discovery rate
FPKM: Fragments per kilobase of transcript per million mapped reads
logFC: Log fold change
RSEM: RNA-Seq by expectation-maximization
NB-LRR: Nucleotide-binding leucine-rich repeat
MLO: Mildew locus O
NB-ARC: Nucleotide-binding APAF-1, R proteins and CED-4 protein
RT-qPCR: Quantitative reverse transcription polymerase chain reaction
HR: Host resistance
PMR: Powdery mildew resistance
PMS: Powdery mildew susceptible
AUDPC: Area under disease progress curve
HPR: Host plant resistance
QC: Quality control
NGS: Next generation sequencing
EST: Expressed sequence tag
ORF: Open reading frame
PE: Paired-end
DR: Disease resistance
DEG: Differentially expressed gene
