# Supplementary material for: Sequencing and analysis of gerbera daisy leaf transcriptomes reveal disease resistance and susceptibility genes differentially expressed and associated with powdery mildew resistance
Source: BMC Plant Biol. 2020 Nov 30;20:539. doi: 10.1186/s12870-020-02742-4 (PMC7706040; doi:10.1186/s12870-020-02742-4)
Supplement: Supplementary file 2 — Additional file 2: Fig. S1. Gerbera breeding lines 06–245-03 (left) susceptible to powdery mildew (PM) and UFGE 4033 (right), resistant to PM used for RNA-sequencing. Fig. S2. Powdery mildew (PM) symptoms in gerbera A) Whole plant infected with PM B) White fungal spores on the adaxial leaf surface C) White PM spores on capitulum D) White PM spores observed on the peduncle and lower flower surface E) PM conidia as observed under a microscope with a 40x objective. Fig. S3. Functional annotation analysis of gerbera RNA-seq data of powdery mildew resistance and susceptible breeding lines using Blast2GO. Fig. S4. Top-hits species distribution of gerbera transcriptome by comparing gerbera transcripts to the viridiplantae database using Blast2GO. Fig. S5. Histogram showing the frequency distribution of gerbera transcripts with which the number of Gene Ontology (GO)-terms are associated. The figure was created using Blast2GO analysis. Fig. S6. Annotation of Gene Ontology-terms assigned to the gerbera transcriptome using WEGO2.0 analysis. Fig. S7. Frequency distribution of enzyme class distribution of gerbera transcripts using Blast2GO. Fig. S8. Frequency distribution of Gene Ontology (GO)-Terms that were enriched among the differentially expressed gerbera transcripts. [file 12870_2020_2742_MOESM2_ESM.docx]

Supplementary Material

# Supplementary Figures


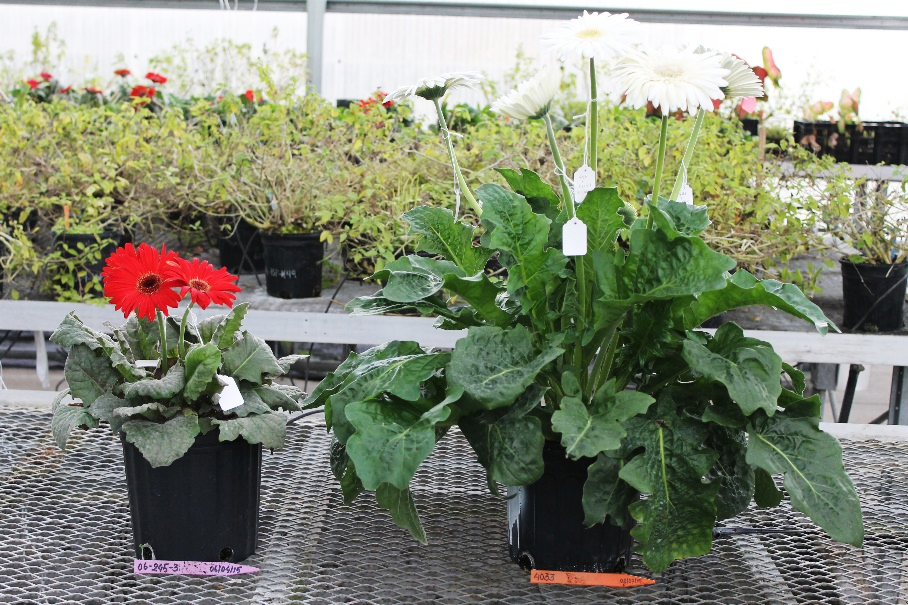


**Supplementary Figure S1**. Gerbera breeding lines 06-245-03 (left) susceptible to powdery mildew (PM) and UFGE 4033 (right), resistant to PM used for RNA-sequencing


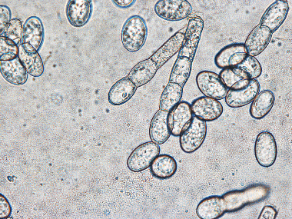

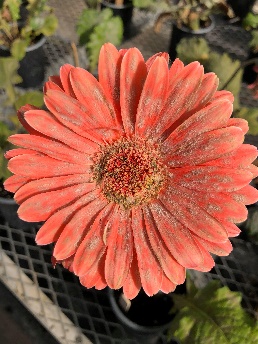

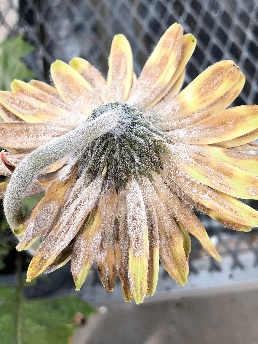

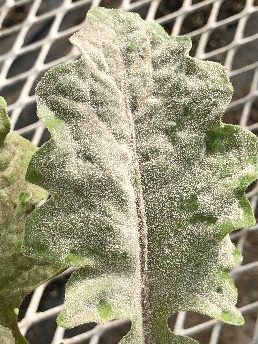

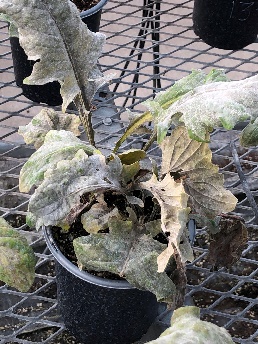


A.

B.

C.

D.

E.

**Supplementary Figure S2**. Powdery mildew (PM) symptoms in gerbera A) Whole plant infected with PM B) White fungal spores on the adaxial leaf surface C) White PM spores on capitulum D) White PM spores observed on the peduncle and lower flower surface E) PM conidia as observed under a microscope with a 40x objective


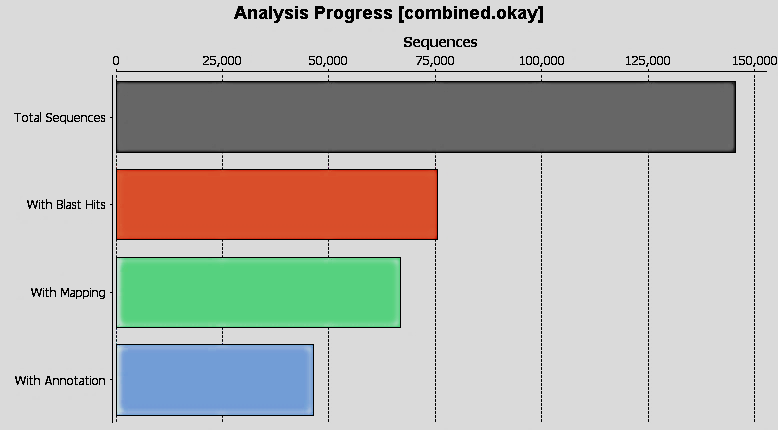


**Supplementary Figure S3**. Functional annotation analysis of gerbera RNA-seq data of powdery mildew resistance and susceptible breeding lines using Blast2GO


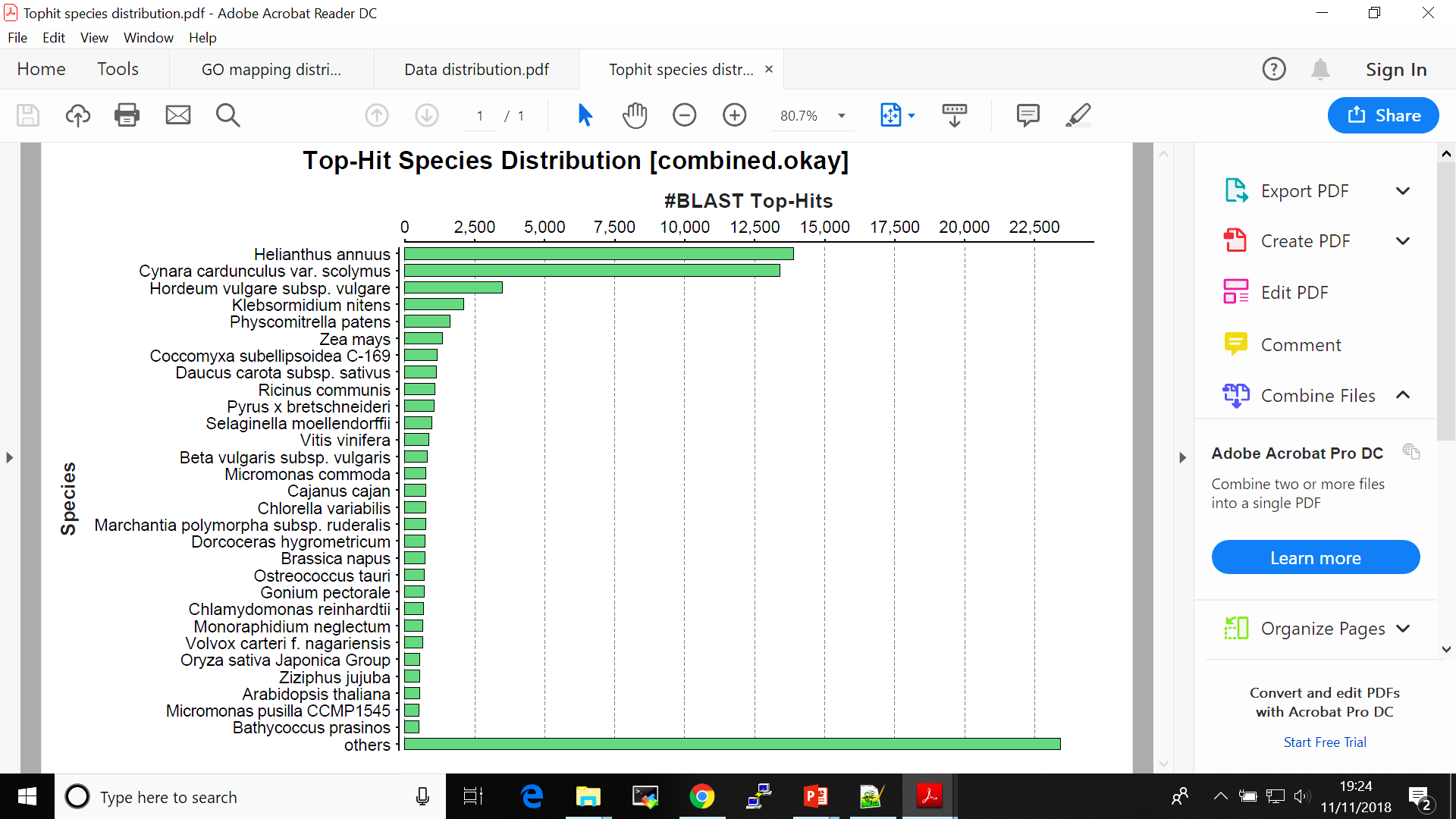


**Supplementary Figure S4**. Top-hits species distribution of gerbera transcriptome by comparing gerbera transcripts to the viridiplantae database using Blast2GO


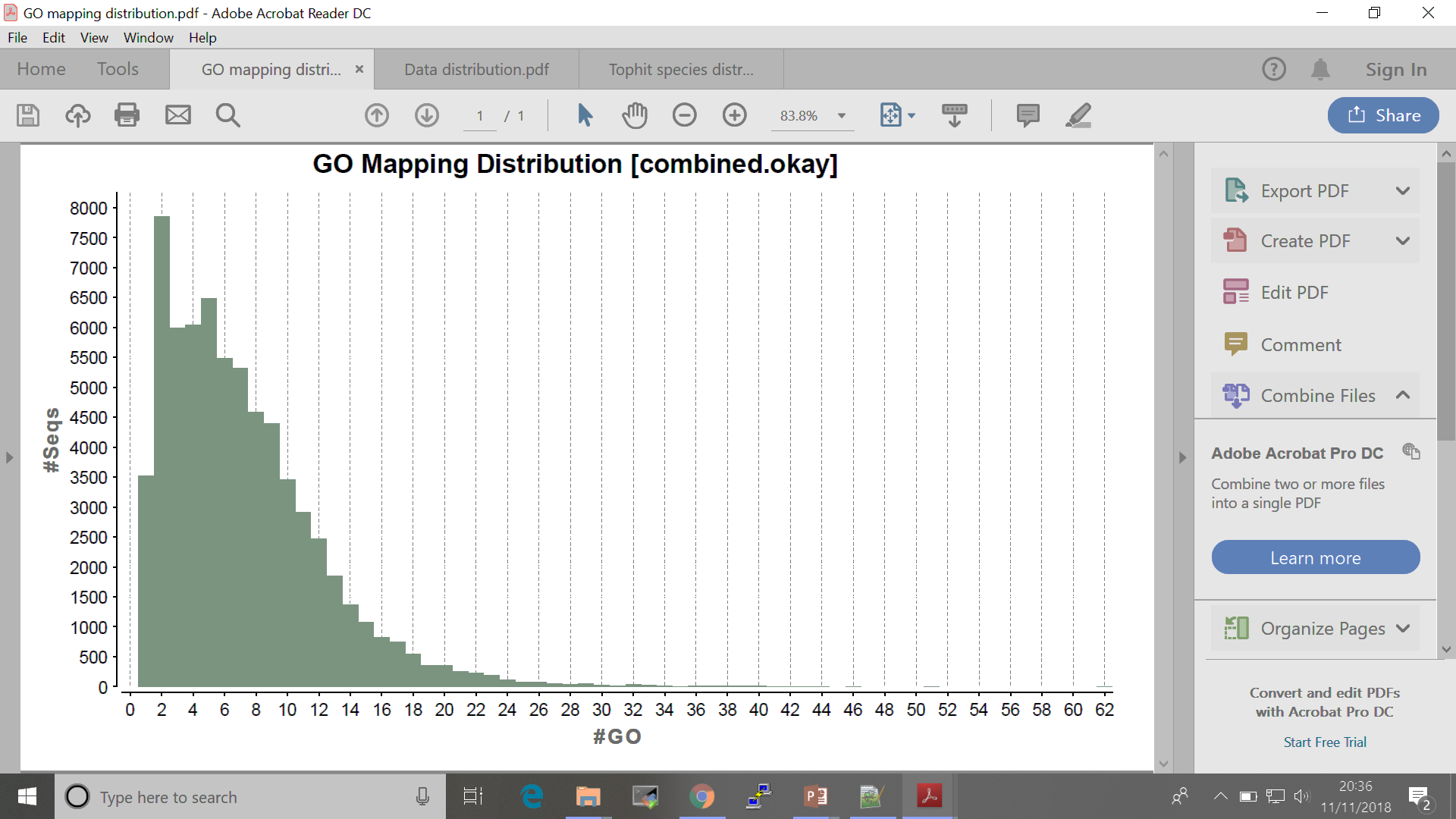


**Supplementary Figure S5**. Histogram showing the frequency distribution of gerbera transcripts to which the number of Gene Ontology (GO)-terms are associated with. The figure was created using Blast2GO analysis


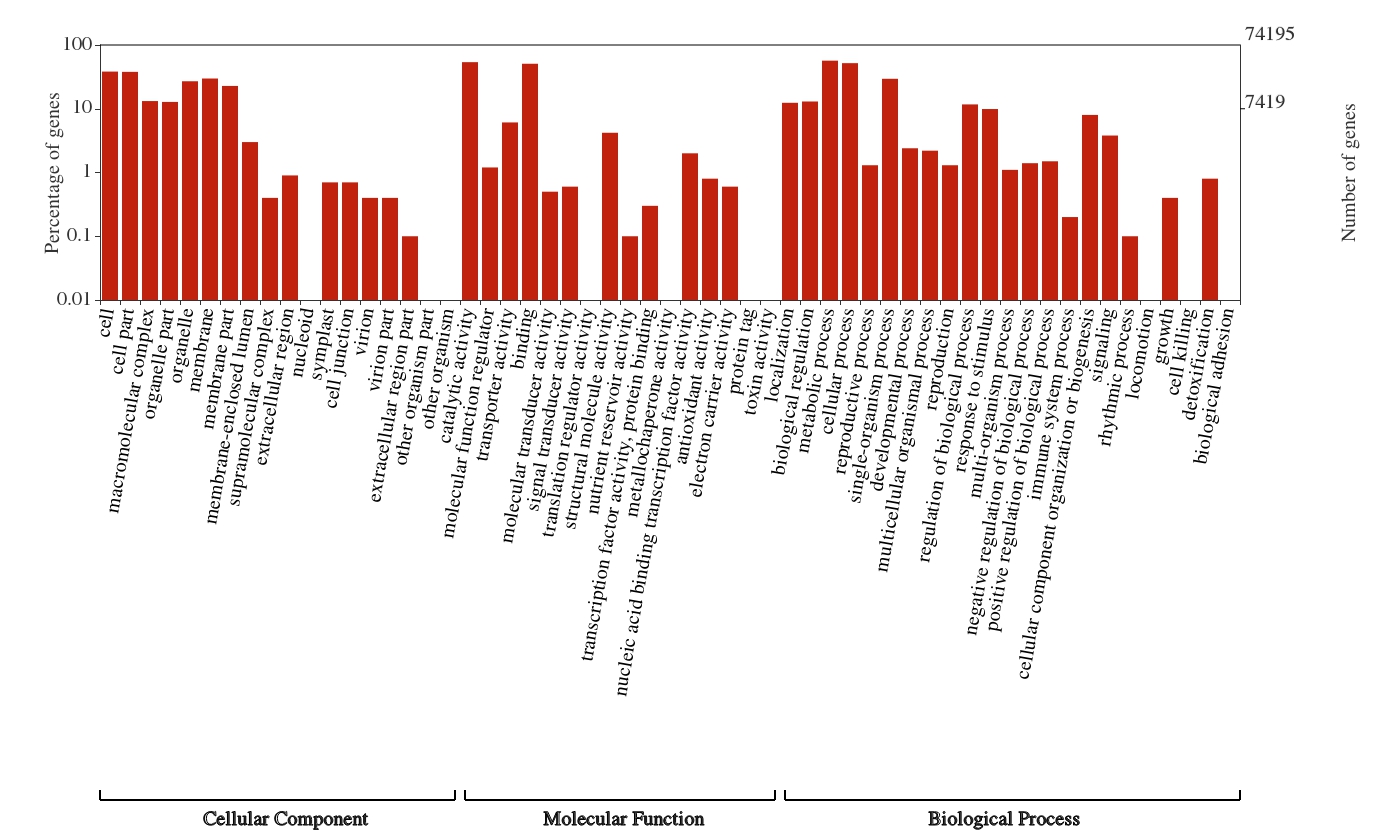


**Supplementary Figure S6**. Annotation of Gene Ontology-terms assigned to the gerbera transcriptome using WEGO2.0 analysis


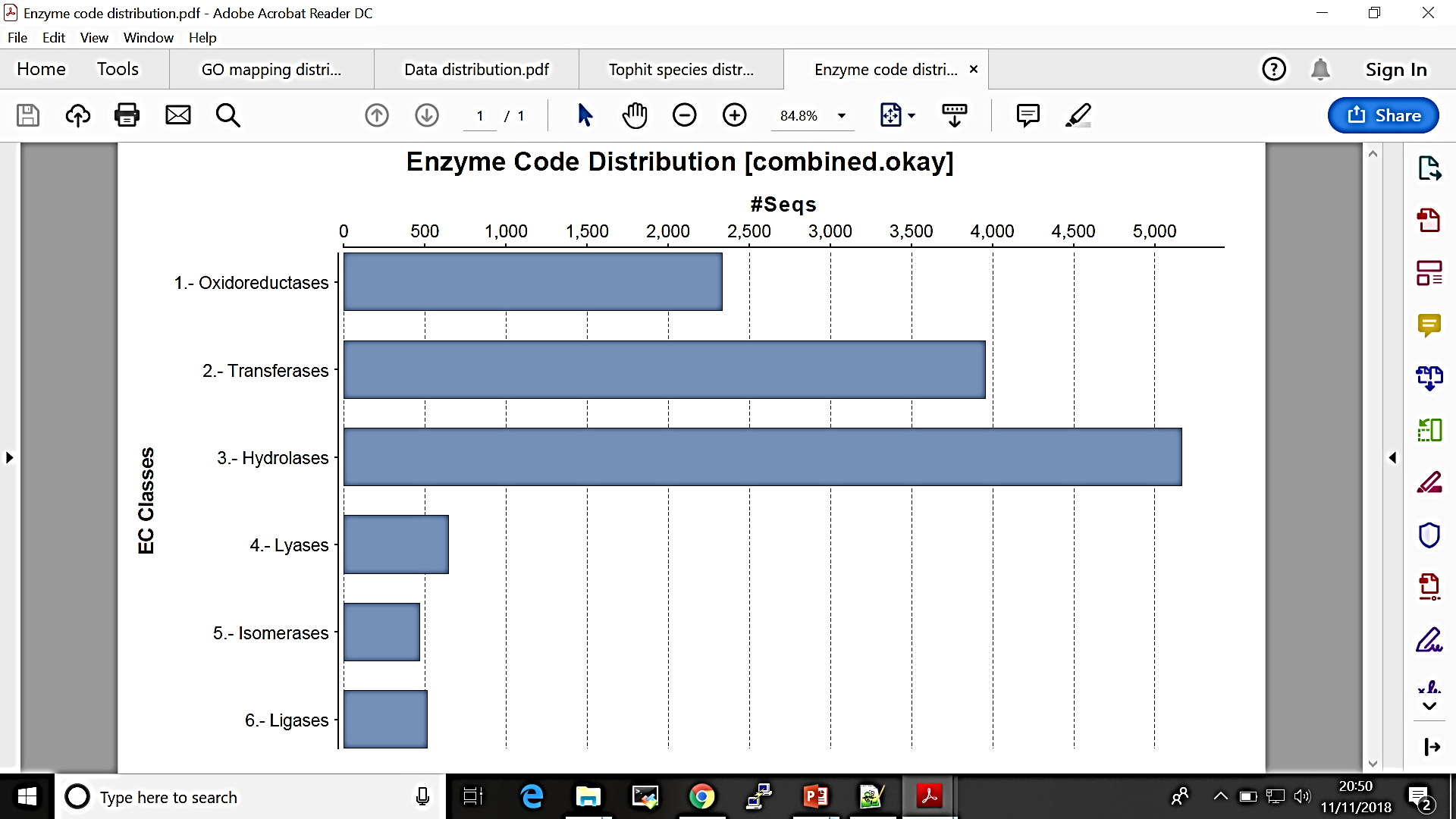


**Supplementary Figure S7**. Frequency distribution of enzyme class distribution of gerbera transcripts using Blast2GO


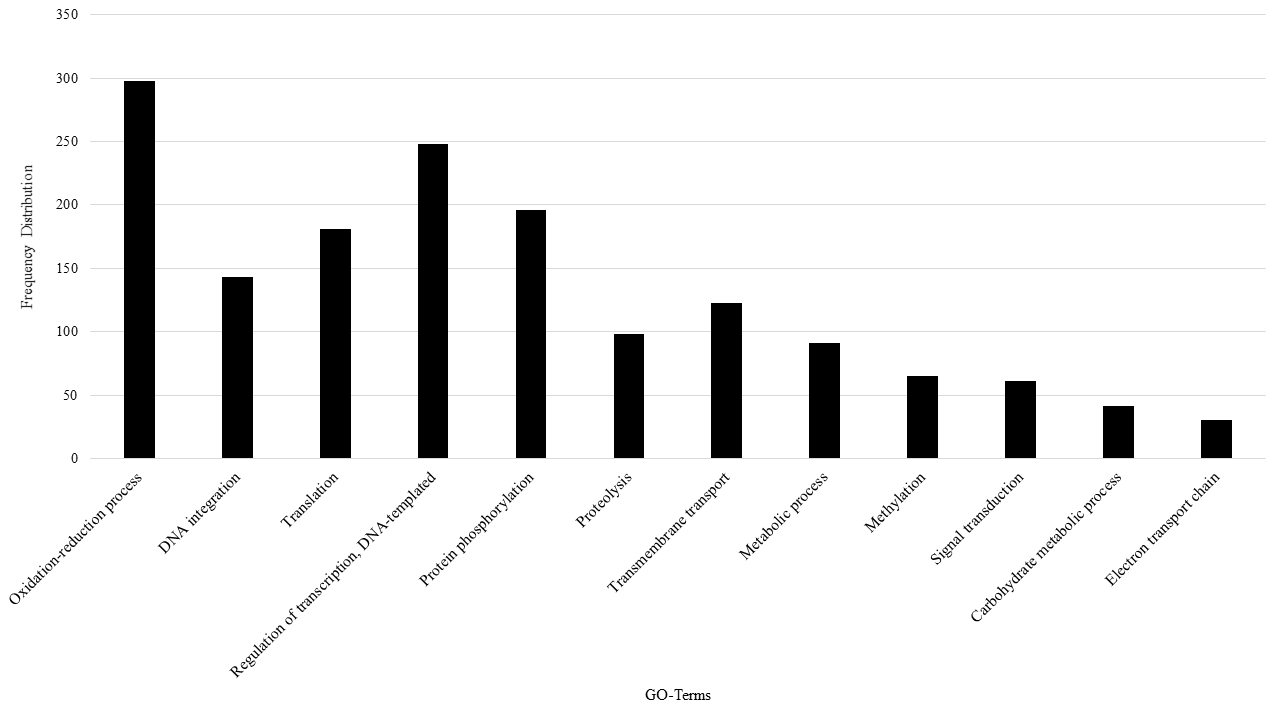
**Supplementary Figure S8**. Frequency distribution of Gene Ontology (GO)-Terms that were enriched among the differentially expressed gerbera transcripts
